# Intraspecific Variation among Social Insect Colonies: Persistent Regional and Colony-Level Differences in Fire Ant Foraging Behavior

**DOI:** 10.1371/journal.pone.0133868

**Published:** 2015-07-21

**Authors:** Alison A. Bockoven, Shawn M. Wilder, Micky D. Eubanks

**Affiliations:** Department of Entomology, Texas A&M University, College Station, Texas, United States of America; Arizona State University, UNITED STATES

## Abstract

Individuals vary within a species in many ecologically important ways, but the causes and consequences of such variation are often poorly understood. Foraging behavior is among the most profitable and risky activities in which organisms engage and is expected to be under strong selection. Among social insects there is evidence that within-colony variation in traits such as foraging behavior can increase colony fitness, but variation between colonies and the potential consequences of such variation are poorly documented. In this study, we tested natural populations of the red imported fire ant, *Solenopsis invicta*, for the existence of colony and regional variation in foraging behavior and tested the persistence of this variation over time and across foraging habitats. We also reared single-lineage colonies in standardized environments to explore the contribution of colony lineage. Fire ants from natural populations exhibited significant and persistent colony and regional-level variation in foraging behaviors such as extra-nest activity, exploration, and discovery of and recruitment to resources. Moreover, colony-level variation in extra-nest activity was significantly correlated with colony growth, suggesting that this variation has fitness consequences. Lineage of the colony had a significant effect on extra-nest activity and exploratory activity and explained approximately half of the variation observed in foraging behaviors, suggesting a heritable component to colony-level variation in behavior.

## Introduction

Individuals vary within populations in many ecologically important ways [1–4] and there is mounting evidence that this variation can have large effects on populations and communities [5–7]. Consistent individual variation in behavior (i.e., personality and behavioral syndromes) may be particularly important in determining the outcomes of inter and intraspecific interactions [8,9]. In terrestrial ecosystems, social insects are often abundant and provide critically important ecosystem functions [10]. Social insects (such as ants and many species of bees and wasps) are the most important pollinators of flowering plants, act as major seed predators and dispersers, prey on agricultural pests and other arthropods, and are major ecosystem engineers that alter soil aeration and nutrient content [10–12]. Despite the pervasive ecological importance of social insects, very little is known about colony-level variation in their behaviors. The goal of this study is to assess colony-level variation in the foraging behavior of an ecologically dominant invasive social insect: the red imported fire ant (*Solenopsis invicta;* Hymenoptera: Formicidae).

Among social organisms there is substantial evidence that within-colony variation in traits such as foraging behavior can increase colony fitness [13–17]. Such variation may extend the behavioral range of the colony and allow better and more rapid response to environmental changes. For example, in harvester ants, workers from different patrilines vary in the time of day they begin foraging, resulting in increased seed collection in colonies with more patrilines [15]. Colonies of social spiders maintain different compositions of “aggressive” or “docile” type individuals and these colony-characteristic ratios have differential success across environments [18]. Such examples demonstrate the ability of intra-group variation to create group-level differences in collective behavior that alter ecological interactions. Surprisingly, behavioral variation at this colony level (differences among colonies) has been much less well studied. Consequently, the extent, persistence, and potential consequences of variation among colonies of social animals are poorly understood. Group differences have been shown to affect heterospecific interactions in colonies of social spiders and alter social dynamics and group outcomes in water striders [19,20]. Among ants, research-to-date on colony-level behavioral variation has been limited to only a scattering of species from out of the hundreds of ant genera (e.g. *Myrmica*: [21]; *Pogonomyrmex*: [22]; *Linepithema*: [23]; *Temnothorax*: [24]). For example, recent work on the harvester ant *Pogonomyrmex barbatus* found that colonies differ in the baseline rates at which foragers leave the nest, and also differ in their behavioral plasticity—specifically the degree to which they adjust their foraging activity based on outside stressors [25]. Daughter colonies may exhibit similar behavior to their mother colonies, indicating a potential heritable component [26]. If colonies consistently vary in foraging behavior and other important traits, then quantifying and understanding colony-level variation will be critical to accurately predict the effects of social insects on interacting species [19,27–29].

We predict that foraging behavior of fire ant colonies will vary significantly. For social insects, as with most animals, foraging occupies a large portion of their lifespan, is vital for growth, reproduction, and survival; yet, it carries some of the greatest risks they will face [30,31]. Foraging may require entering dangerous or unknown environments, may attract or expose organisms to predators, and may place organisms in direct or indirect competition with others [32]. Animals must balance the potential costs of risky behaviors against other fitness needs [33]. If foraging is risky or energetically costly, then we would predict that fire ant colonies will show evidence of trade-offs between minimizing risk and energy expenditure (e.g. extra-nest activity and foraging effort) and maximizing food collection and colony growth.

We tested for the existence and extent of variation in foraging behavior in natural populations of the red imported fire ant by quantifying colony and regional-level variation. We quantified variation in ground and arboreal foraging, the persistence of variation over five weeks and across two microhabitats, and quantified trade-offs in fire ant foraging and colony growth by regressing foraging activity, food collection, and colony biomass. We also estimated the broad sense heritabilities of foraging behavior using single-lineage colonies.

## Methods

### Study System

Fire ants are an invasive pest species across much of the southern United States and many other areas around the world [34]. As such, they have significant ecological, economic, and health consequences [35]. Like most ants, fire ants forage by sending worker scouts into their territory to locate resources. These scouts return to the nest or to nearby foraging tunnels and recruit other workers to the resource using pheromone trails [35]. We selected collection sites in Texas and Mississippi because in previous field work we observed differences in the arboreal and ground-level foraging behavior of fire ants from the two regions [36]. This is of particular interest because differences in the use of arboreal resources have been linked to the invasive success of fire ants in the United States and the ecological dominance of multiple ant species [37–41].

### Experiment 1: Colony and regional-level variation

#### Field colony collection and maintenance

We collected red imported fire ant colonies from Texas (Texas A&M Field Laboratory, Burleson Co., TX; 30° 33' 14"N, 96° 25' 41"W; permission granted by Texas A&M Agrilife Research) and Mississippi (Homochitto National Forest, Amite Co., MS; 31° 16’ 11”N 91° 08’ 14”W; permission granted by Mississippi Dept. of Wildlife, Fisheries & Parks) in order to quantify colony and regional-level variation in foraging behavior. We extracted colonies from soil using drip floatation [42] and used each field colony (colony of origin) to create two standardized experimental colonies of 2 queens, 50 brood, and 1 gram of workers (~2000 ants) which served as colony replicates. Only field colonies found to contain multiple queens were included in this experiment (TX n = 17, MS n = 16). Each experimental colony was placed in a fluon-lined (Insect-a-slip Insect Barrier, BioQuip Products, 2321 Gladwick St., Rancho Dominguez, CA) foraging arena (38x55x6cm) containing an artificial nest dish (15cm diameter black-lidded petri dish with dampened plaster) and water tube.

Throughout the experiment, experimental colonies were maintained in standardized laboratory conditions (temperature 24–32°C, 40–70% humidity, 12:12 light/dark cycle) and fed three times per week, alternating between two 3 mL tubes of artificial nectar [43] and one male and one female adult cricket, *Acheta domesticus*. By observing the foraging behavior of standardized colonies in the lab, we controlled for variation due to environment and colony size and ratio of brood to workers. All food was removed from the foraging arenas 24hrs prior to behavioral assays and all assays were conducted at a standardized time (10AM).

#### 1a) Survey of colony and regional-level variation in foraging behavior of natural populations

To quantify variation in ground-level foraging among standardized experimental colonies, we placed a freshly killed cricket in the foraging arena, 30 cm from the artificial nest, and recorded the number of ants present at the cricket after 10 minutes, and then every 30 minutes for 150 minutes. We also observed colonies every minute for 10 minutes and then at 30 minute intervals to determine time to discovery of resource and time to formation of a visible trail of recruiting ants. Colonies which had not discovered or formed a trail to the resource within the observation period were scored with the final time value. The following day we assayed variation in climbing behavior by recording discovery, trail formation, and recruitment to an elevated cricket placed at the top of a 30 cm dowel placed 30 cm from the artificial nest. Observations were made as above, with an additional final observation at 330 minutes. At the end of the week, we measured extra-nest activity by counting the number of ants active outside the nest in the foraging arena three times and taking the average. We measured exploratory activity by introducing a novel climbing structure comprised of two halves of a 7.6 x 12.7 cm index card skewered vertically at the top of a 30 cm bamboo skewer (S1 Fig). We then counted the number of ants exploring the structure at 20 minute intervals for 2 hours and took the average of these counts.

The behavioral data from the colony replicates were used to compare variation at both the level of region (Texas vs. Mississippi populations) and colony of origin. We used multiple regression with region and colony of origin (nested in region) as covariates in the model to determine the effects of these variables on variation in the activity, exploration, and ground-level and elevated foraging recruitment traits. Count data were square-root transformed. All results in this study were analyzed using SAS v. 9.3 (SAS Institute Inc., Cary, NC). Data available from the Dryad Digital Repository (doi:10.5061/dryad.94r7j).

#### 1b) Persistence of variation (before and after exposure to different foraging habitats)

After one week of equilibration to laboratory conditions and one week of foraging assays as described above, we divided the experimental colonies into treatments of two different foraging habitats in order to determine if behavioral variation would persist across exposure to different environmental complexities and foraging contexts. Each colony of origin was represented in each treatment group by one standardized experimental colony. In the first treatment, we provided all colonies with six 30-cm upright wooden dowels and all food items were placed at the top of two randomly-selected dowels throughout the experiment (“elevated” foraging habitat), requiring ants to climb and forage in a more complex environment. In the second treatment, we placed all food items at ground level, next to horizontal wooden dowels (“ground-level” foraging habitat). Ants were maintained in these conditions for five weeks. In the following week we temporarily removed all elevated foraging structures and then assayed the behavior of all colonies as described previously, first in the ground-level foraging habitat and then in the elevated foraging habitat.

We used repeated measures analysis of variance to compare the behavioral variables (discovery, trail formation, and recruitment to a ground-level or elevated cricket; extra-nest activity; exploration) before and after exposure to different habitats. Foraging habitat treatment, region, and colony (nested in region) were included as covariates in the model, and count data were square-root transformed. We analyzed within-subjects effects using the more conservative multivariate analysis of variance which does not assume sphericity of variance. Interaction terms that were not significant were sequentially excluded from the model.

#### 1c) Food collection and colony growth

We measured the weight of cricket collected by each colony during foraging assays at the beginning, middle, and end of the experiment. We weighed freeze-killed crickets prior to foraging assays, allowed fire ant colonies to forage on them for 24 hours, and then removed and dried unconsumed crickets at 60°C for 24 hours before measuring the dry weight cricket remaining. The dry weight collected by fire ants was estimated using previously established methods, by comparison to a control set of unconsumed crickets weighed wet and dry [43]. Upon completion of the experiment, we collected and dried all adults and brood at 60°C for 24 hours before weighing them. The final dry weight of each colony was used to compare colony growth.

We used repeated measures analysis of variance as previously described to test for the effects of foraging habitat, region, and colony (nested in region) on dry weight of cricket collected over time. We used multiple regression to analyze the effects of foraging habitat, region, and colony (nested in region) on final colony size. To determine correlations between weight of food collected, final colony size, and colony behaviors (as first measured) we calculated Pearson’s product-moment coefficients.

### Experiment 2: Single-lineage colony experiments

#### Single-lineage colony collection and maintenance

In order to establish colonies with a minimum of environmental and within-colony genetic variance, we collected newly-mated foundress queens and reared single-lineage colonies in a standardized laboratory environment. Invasive fire ant colonies may be monogyne or polygyne (having a single queen or multiple, unrelated queens in a nest), creating the potential for many genetic lineages in a single field colony. Fire ant queens mate monandrously (or primarily monandrously) so that a single queen produces only workers from a single patriline, or genetic lineage [35,44]. Fire ants mate in nuptial flights high in the air and attempts to artificially cross them in the lab have proved challenging and largely ineffective [45,46]. Thus, studies of heritability must be approached via indirect methods. Foundresses were collected after two mating flights in College Station, TX (30° 36' 54"N, 96° 20' 60"W) and Conroe, TX (30° 14' 5"N, 95° 28' 8"W) and cloistered individually in darkened nest tubes (permission for collection was obtained from Texas A&M Agrilife and owners of private land). Seven days after the first worker eclosed, we moved colonies into standard artificial nest dishes and arenas, as previously described for field-collected colony maintenance, and maintained all colonies in standardized environmental conditions on a standard diet: water *ad libitum*, 14mL artificial nectar replaced weekly, and up to two crickets provided three times weekly. Up to four additional nest dishes were added over time. All colonies were at least six months old prior to the experiment.

#### 2) Variation among single-lineage colonies

In order to estimate the contribution of lineage to intraspecific variation in fire ant foraging behavior, we created three standardized experimental colonies each composed of 0.65g workers (~1300 ants) and about 100 brood for each of 15 single-lineage colonies and assayed their foraging behavior. Workers were collected randomly from disturbed ants both inside and outside the artificial nest in order to ensure a representative selection of all task-groups. The experimental colonies were maintained in individual trays under standardized conditions. Colonies were assayed as previously described for extra-nest activity, exploratory activity, and time to discovery and number of ants recruiting to a single cricket placed at ground-level 30cm outside the nest-entrance. The number of ants at the cricket was recorded after five minutes and then every ten minutes for 60 minutes.

The behavioral data of the single-lineage colonies were analyzed as described for field colonies, using multiple regression to test for effects of region and colony (nested in region). Queen mating flight of origin had no effect and was excluded from the model. The R^2^ value of the model was used to estimate the percentage of variation explained by colony of origin, a rough estimate of broad-sense heritability [47,48].

## Results

### 1a) Colony and regional-level variation in foraging behavior of natural populations

**Fig 1:**
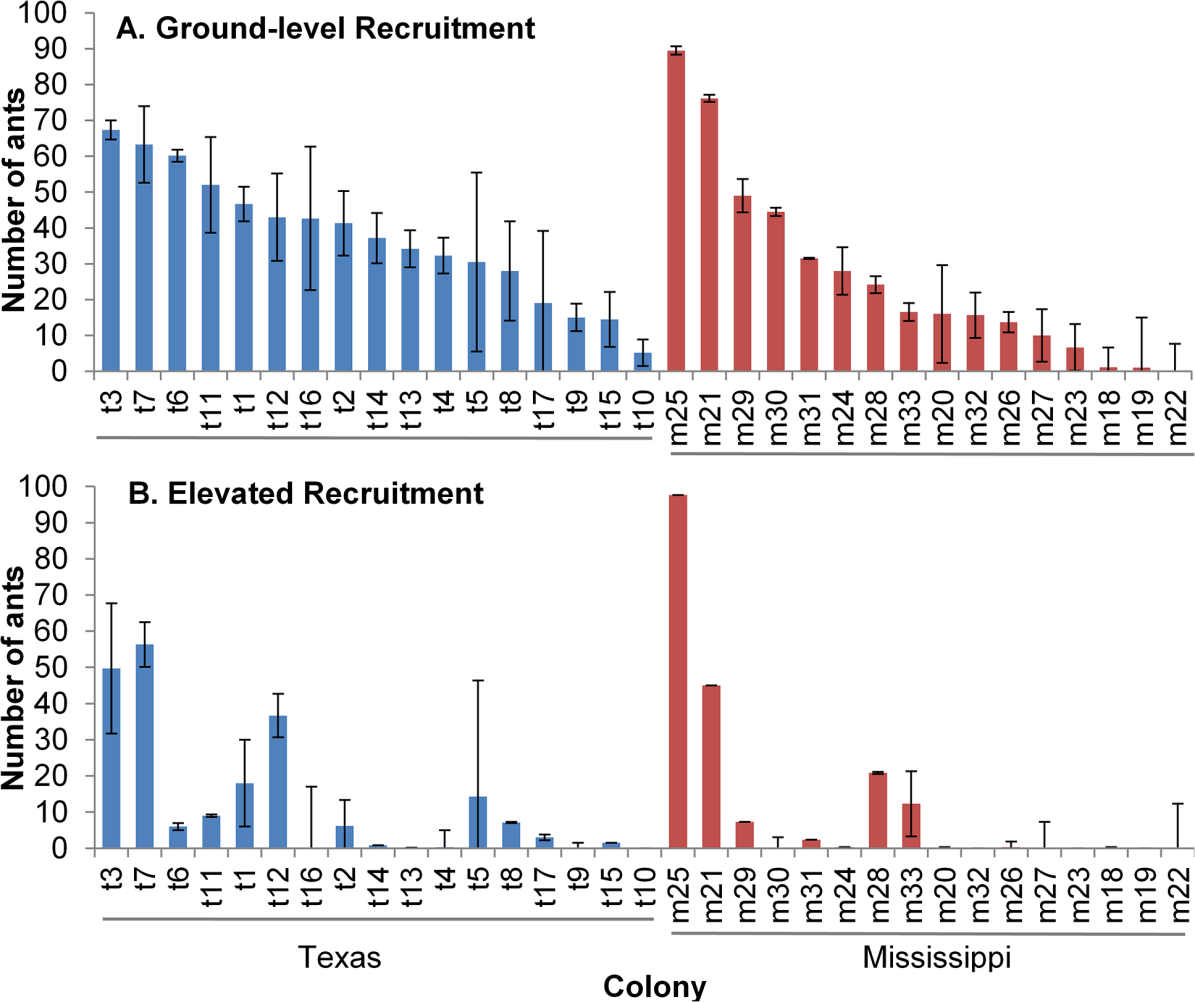
Average number of fire ants recruiting to ground-level or elevated crickets. Crickets were placed at (A) ground-level or (B) 30cm up a wooden dowel, prior to experimental treatment. Each bar represents average recruitment for a single colony of origin (n = 2); error bars show standard error.

We observed significant variation among fire ant colonies in extra-nest activity (F_31,33_ = 3.93, p = <0.0001), exploratory activity (F_31,33_ = 1.87, p = 0.0405), and recruitment to ground-level (F_31,33_ = 4.63, p<0.0001) and elevated food (F_31,33_ = 5.08, p<0.0001). Behavioral variation among colonies was often large. For example the most active colonies recruited more than 40 times more workers to crickets on average than the least active colonies (Fig 1). When foraging at ground-level, the number of ants recruited to crickets varied significantly by colony of origin (Fig 1A) as did recruiting trail formation (F_31,33_ = 2.43, p = 0.0069), but not discovery time (F_31,33_ = 0.92, p = 0.5907). Colony-level variation in foraging behavior at elevated foods was even more pronounced, with a highly significant effect of colony of origin (nested in region) for all measured variables (Fig 1B: discovery: F_31,33_ = 3.16, p = 0.0008; trail: F_31,33_ = 7.51, p = <0.0001).

**Fig 2:**
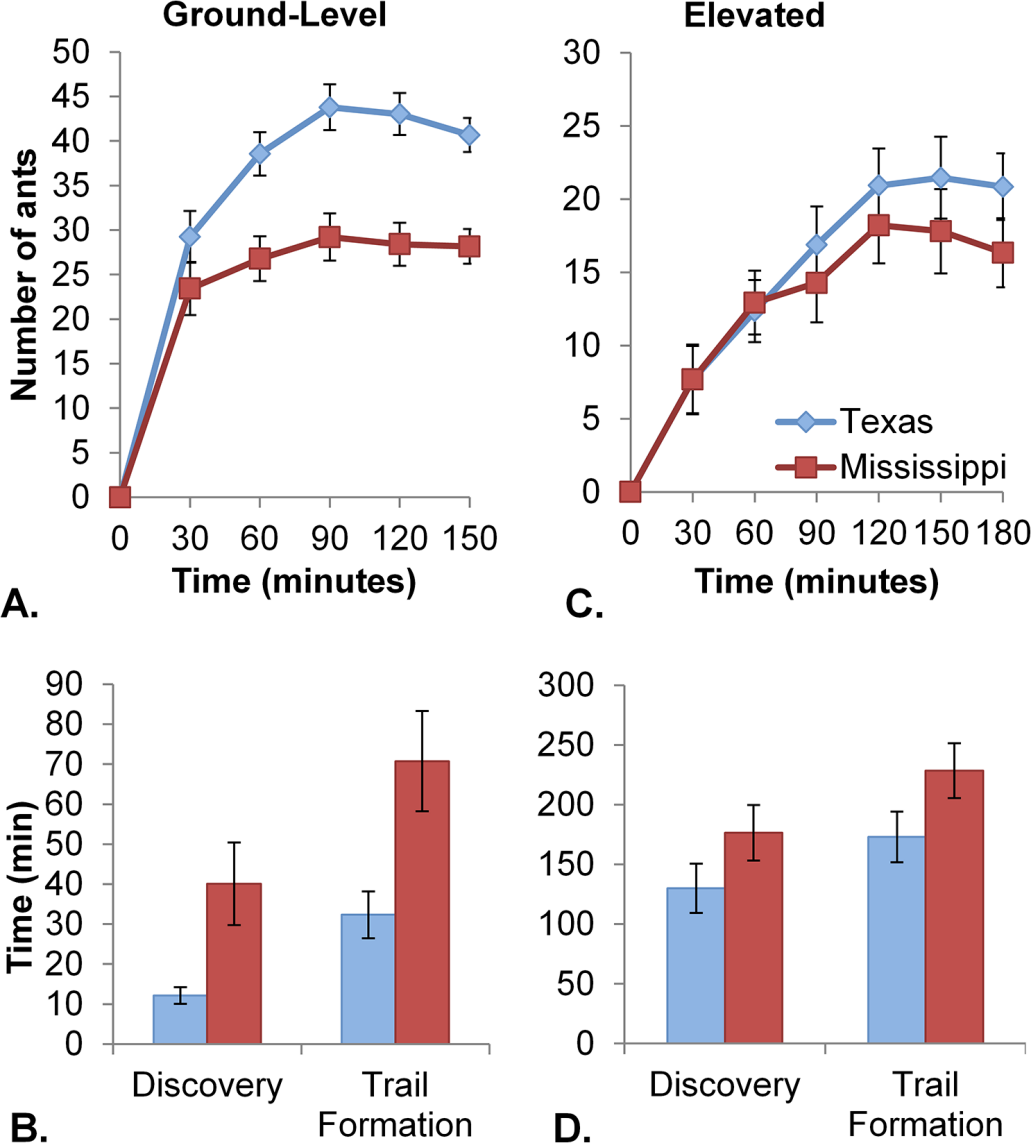
Regional differences in recruitment of fire ants to ground-level or elevated crickets. Colonies were collected in Texas (light diamond, n = 17) or Mississippi (dark square, n = 16) and crickets were placed at ground-level (A and B) or 30cm up a wooden dowel (C and D). (A) and (C) show average number of ants observed at the cricket over time while (B) and (D) show the average time to resource discovery and time to formation of a recruiting trail. Error bars show standard error.

We also observed significant regional differences in ant behavior, which fit our expectations for behavioral patterns in relationship to site invasion history. Fire ants from Texas colonies (closer to the invasion front) recruited to ground-level crickets in significantly higher numbers than ants from Mississippi colonies (closer to the invasion origin) (Fig 2A: F_1,31_ = 17.08, p = 0.0002) with on average 40% more ants foraging at crickets. Ants from Texas colonies also discovered and formed recruiting trails to ground-level crickets significantly faster than those from Mississippi colonies (Fig 2B: discovery: F_1,33_ = 7.17, p = 0.0115; trail: F_1,33_ = 13.53, p = 0.0008). When ants were required to climb 30cm to reach crickets, the regional differences in discovery and trail formation times were similar to those at ground-level, with Texas colonies locating and developing foraging trails to elevated crickets significantly faster than colonies from Mississippi (Fig 2D: discover: F_1,33_ = 4.61, p = 0.0391; trail: F_1,33_ = 13.16, p = 0.0010). Regional patterns of extra-nest and exploratory activity as well as elevated recruitment trended in the same direction as the previous traits, but were non-significant (extra-nest: F_1,33_ = 3.51, p = 0.0700; exploration: F_1,33_ = 1.79, p = 0.1901elevated recruitment: Fig 2C: F_1,33_ = 1.55, p = 0.2226).

### 1b) Persistence of variation (before and after exposure to different foraging habitats)

**Fig 3:**
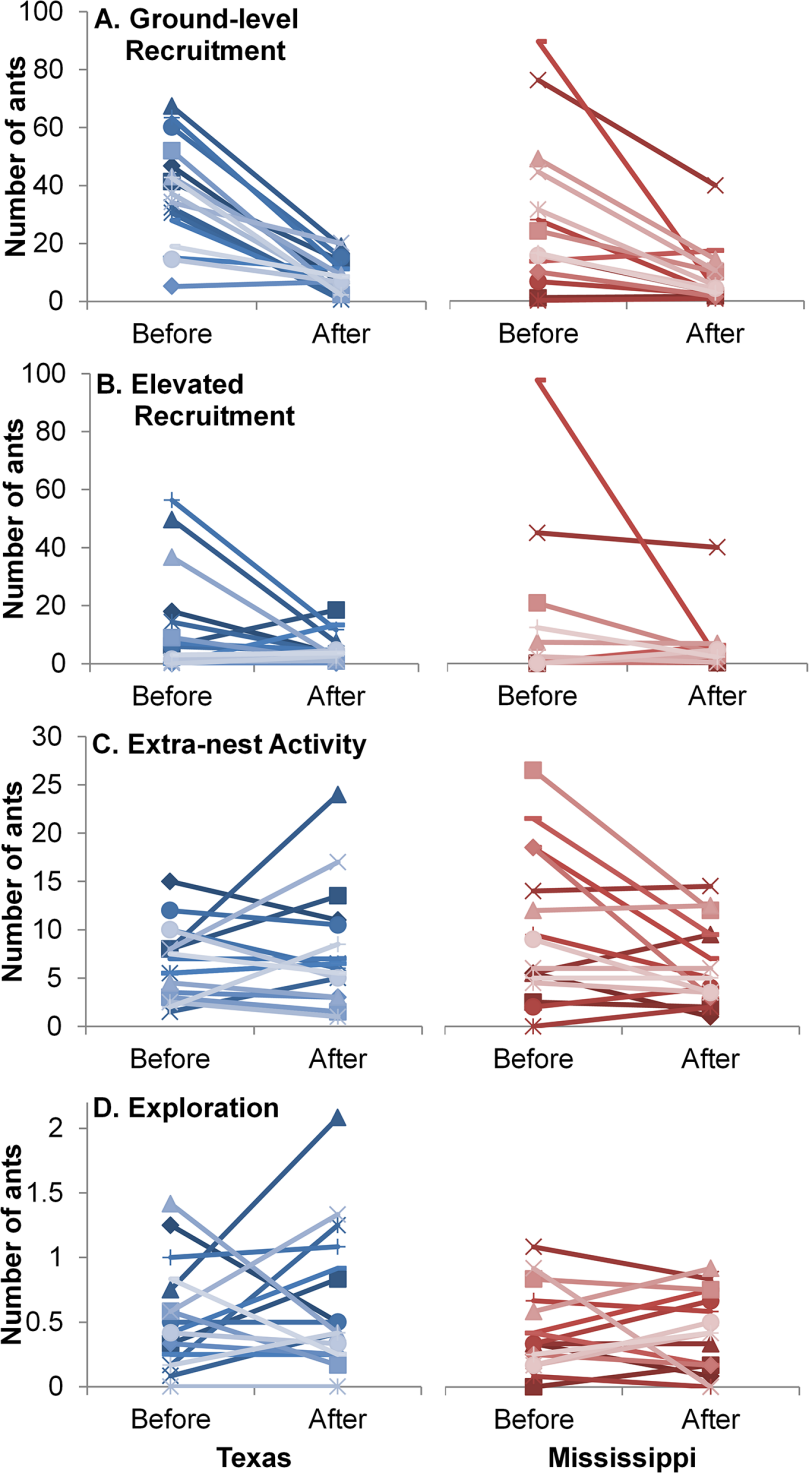
Colony differences in fire ant foraging behavior before and after exposure to different foraging habitats. Graphs show average number of ants (A) at a ground-level cricket, (B) at a cricket elevated 30cm up a wooden dowel, (C) active outside the nest and (D) exploring a novel structure. Each line represents average for a colony of origin (n = 2), for fire ant colonies from Texas (left) and Mississippi (right) before and after five weeks exposure to different foraging habitat treatments.

Colony and regional-level variation in behavior generally persisted over time and across exposure to different foraging habitats. Colony of origin was a significant factor in the variation observed for all measured foraging variables across the five weeks (summarized Table 1; S1 Table; Fig 3A–3D) and the activity and foraging behavior of colonies at the beginning of the experiment was highly correlated with their behavior at the end of the experiment (Pearson’s correlation: extra-nest activity: r = 0.4275, p = 0.0003; exploratory activity: r = 0.3471, p = 0.0043; ground-level recruitment: r = 0.4562, p = 0.0001; elevated recruitment: r = 0.3192, p = 0.0090). Additionally, extra-nest activity, trail formation to elevated resources, and average recruitment to both ground-level and elevated resources of experimental colonies from the same colony of origin tended to increase or decrease over time in a colony-specific manner (Table 1; Fig 3A–3C). Only ground-level recruitment showed a significant effect of time independent of colony effects, with significantly fewer ants on average recruiting to resources at the end of the experiment. Neither foraging habitat (treatment) nor time by treatment effects were significant for any measured traits (Table 1; S1 Table).

**Table 1:** Analysis of behavior before and after exposure to different foraging habitats.

Trait	Effect	p	F	df 1	df 2
**Average recruitment to ground-level cricket (30–90min)**	Foraging Habitat	0.3554	0.88	1	32
	Region	0.0007*	14.11	1	32
	Colony(Region)	< .0001*	4.98	31	32
	Time	< .0001*	202.61	1	32
	Time*F.Habitat	0.6727	0.18	1	32
	Time*Region	0.0015*	12.05	1	32
	Time*Colony(Region)	0.0001*	3.88	31	32
**Average recruitment to elevated cricket (30–90min)**	Foraging Habitat	0.9678	<0.01	1	32
	Region	0.1328	2.38	1	32
	Colony(Region)	< .0001*	5.22	31	32
	Time	0.2443	1.41	1	32
	Time*F.Habitat	0.1431	2.25	1	32
	Time*Region	0.6077	0.27	1	32
	Time*Colony(Region)	0.0002*	3.68	31	32
**Extra-nest Activity**	Foraging Habitat	0.0791	3.29	1	32
	Region	0.9922	<0.01	1	32
	Colony(Region)	0.0002*	3.76	31	32
	Time	0.0835	3.19	1	32
	Time*F.Habitat	0.3572	0.87	1	32
	Time*Region	0.0039*	9.66	1	32
	Time*Colony(Region)	0.0092*	2.35	31	32
**Exploration**	Foraging Habitat	0.0443*	4.38	1	32
	Region	0.0980	2.91	1	32
	Colony(Region)	0.0224*	2.07	31	32
	Time	0.7344	0.12	1	32
	Time*F.Habitat	0.1255	2.48	1	32
	Time*Region	0.9130	0.01	1	32
	Time*Colony(Region)	0.1680	1.41	31	32

Table summarizes repeated measures analysis of variance for standardized experimental colonies in standardized foraging habitats before and after being exposed to different foraging habitats for five weeks. Within subjects effects use multivariate analysis of variance; lambda is converted to the appropriate F value.

(*) denote significance at alpha = 0.05.

**Fig 4:**
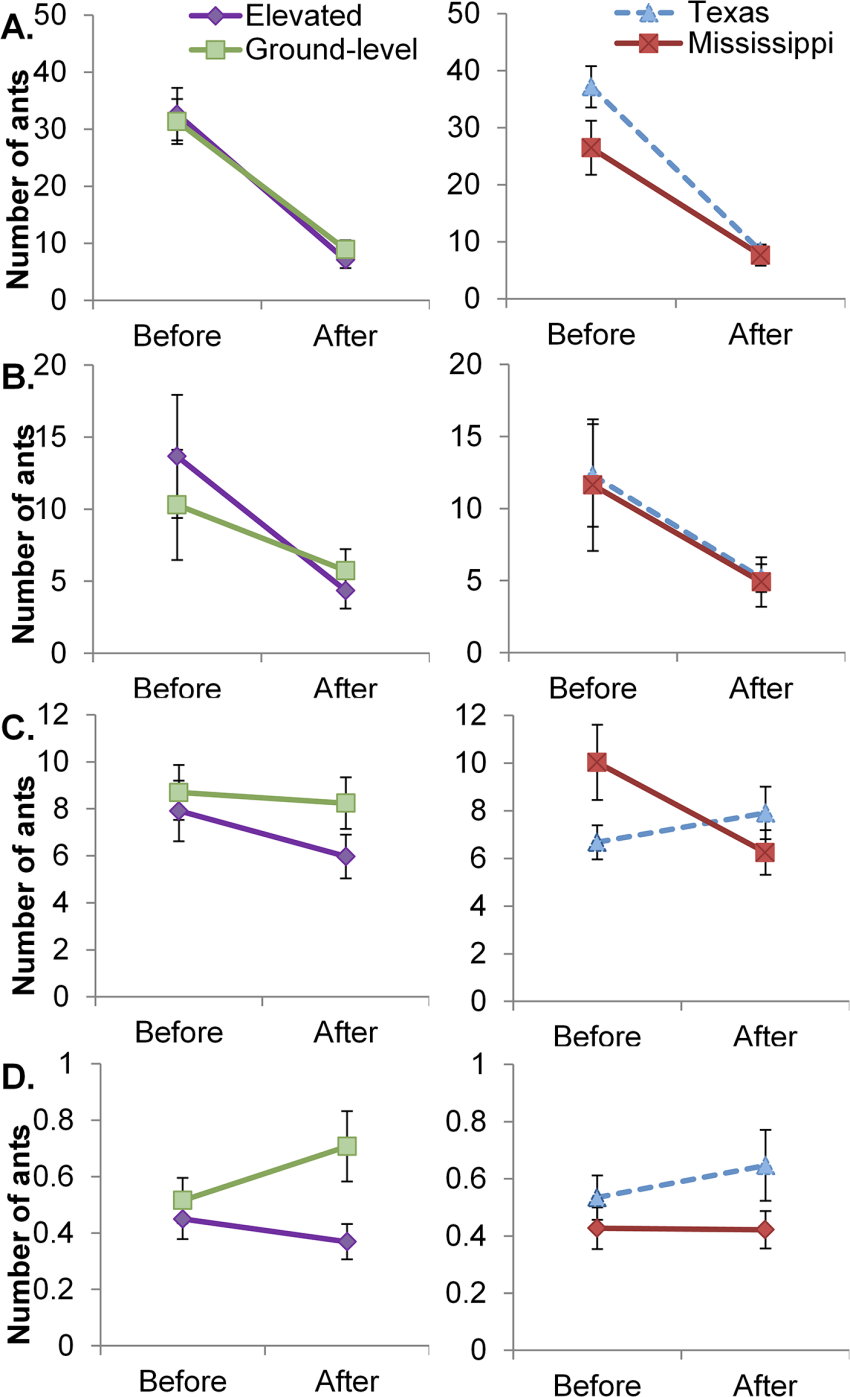
Foraging habitat and regional effects on fire ant foraging behavior. Average number of ants (A) at a ground-level cricket, (B) at a cricket placed 30cm up a wooden dowel, (C) active outside the nest and (D) exploring a novel structure, before and after five weeks exposure to different foraging habitats. Graphs show colonies grouped by foraging habitat treatment (left: elevated, dark diamond (n = 33) vs. ground-level, light square (n = 33)) and by region of origin (right: Texas, light triangle (n = 17) vs. Mississippi, dark square (n = 16)). Error bars show standard error.

Significant regional differences in many foraging behaviors also persisted before and after exposure to different foraging habitats (Table 1; S1 Table: ground recruitment, discovery, and trail; elevated discovery and trail). Fire ants from Mississippi maintained significantly lower average ground-level recruitment than ants from Texas. The ground-level recruitment of Texas ants changed more over time than that of ants from Mississippi, decreasing significantly (Table 1, Fig 4A). Colonies from Mississippi also took longer to discover resources and form recruitment trails than colonies from Texas (S1 Table; S2 Fig). Average recruitment to elevated crickets continued to exhibit no significant regional differences (Table 1, Fig 4B). Over the course of the experiment, Texas ants significantly increased extra-nest activity while Mississippi ants decreased their activity (Table 1, Fig 4C). Ants from different regions did not significantly alter their exploratory activity over time (Table 1, Fig 4D).

### 1c) Food collection and colony growth

**Fig 5:**
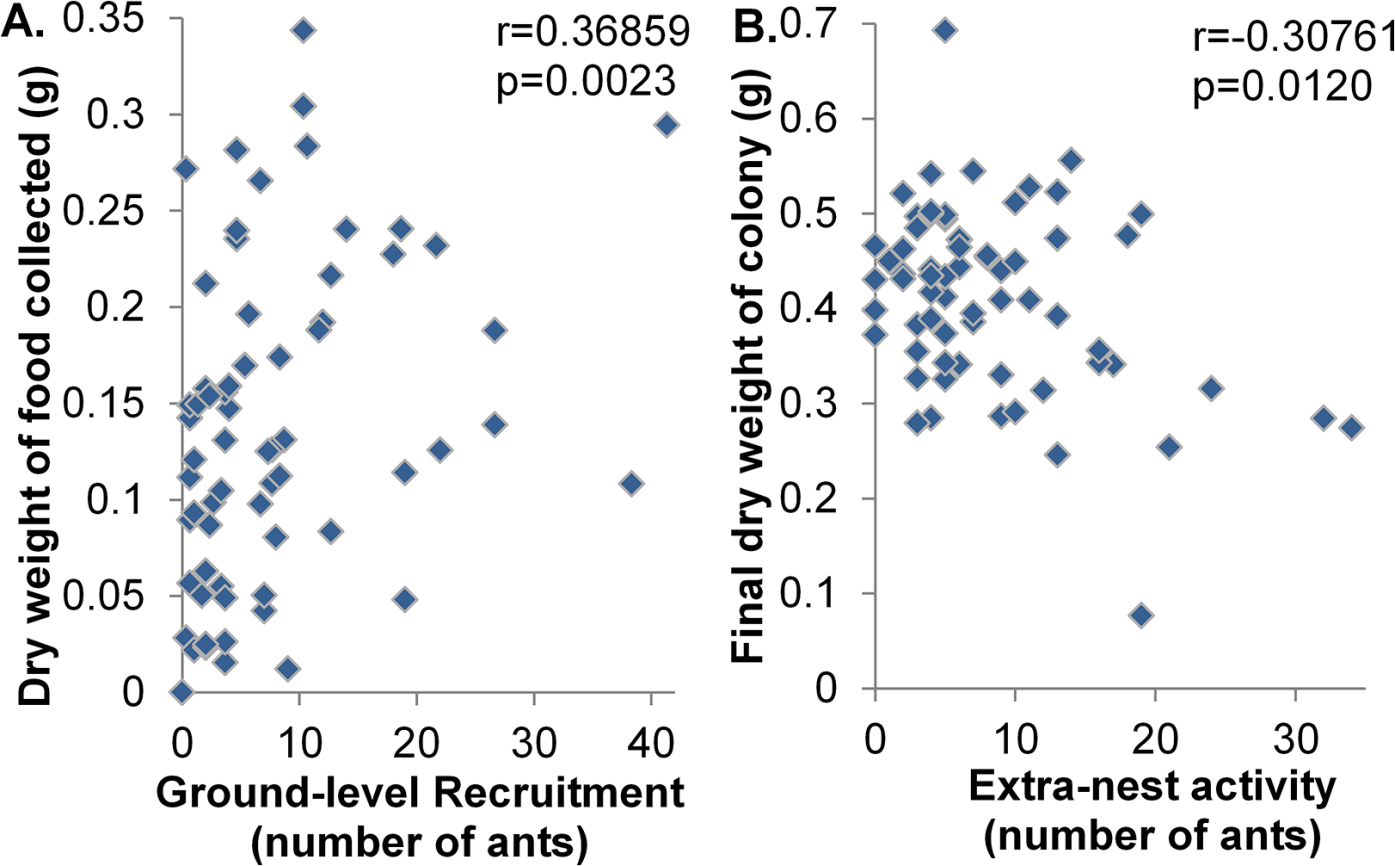
Effects of behavior on fire ant colony performance measures. Graphs show (A) Ground-level recruitment versus colony food collection and (B) extra-nest activity versus colony size (final dry weight of workers and brood).

Although all measured foraging variables were significantly correlated with each other (Pearson’s test p<0.05), we observed differences in their relationships with the amount of food collected by each colony and colony growth. Colonies that discovered and formed recruiting trails to crickets faster, or recruited more ants on average within the first hour also tended to collect a greater average dry weight of cricket over a 24 hour period (Fig 5A; discovery: r = -0.32257, n = 66, p = 0.0083; trail: r = -0.38477, n = 66, p = 0.0014; ground-level: r = 0.36859, n = 66, p = 0.0023; elevated: r = 0.25270, n = 66, p = 0.0014). Colony extra-nest activity and exploration did not correlate with the cricket biomass collected. Ants from Texas collected significantly greater dry weight of crickets than ants from Mississippi, collecting upwards of 50% more cricket by weight by the end of the experiment (F_1,32_ = 6.19, p = 0.0182). Weight of cricket collected was not significantly affected by either foraging habitat or colony of origin, although weight collected increased over time (F_2,31_ = 46.33; p = <0.0001).

Colony replicates from the same colony of origin tended to have similar final colony sizes (F_31,32_ = 3.32, p = 0.0006). Colony size correlated negatively with extra-nest activity (Fig 5B; r = -0.30761, n = 66, p = 0.0120). Colonies with higher activity at the beginning of the experiment had lower final weights, and were as much as three times smaller than less active colonies by the end of the experiment. Final colony weight did not correlate significantly with recruitment or weight of cricket collected and neither exposure to different foraging habitats nor region of origin had a significant effect on final colony size.

### 2) Variation among single-lineage colonies

**Fig 6:**
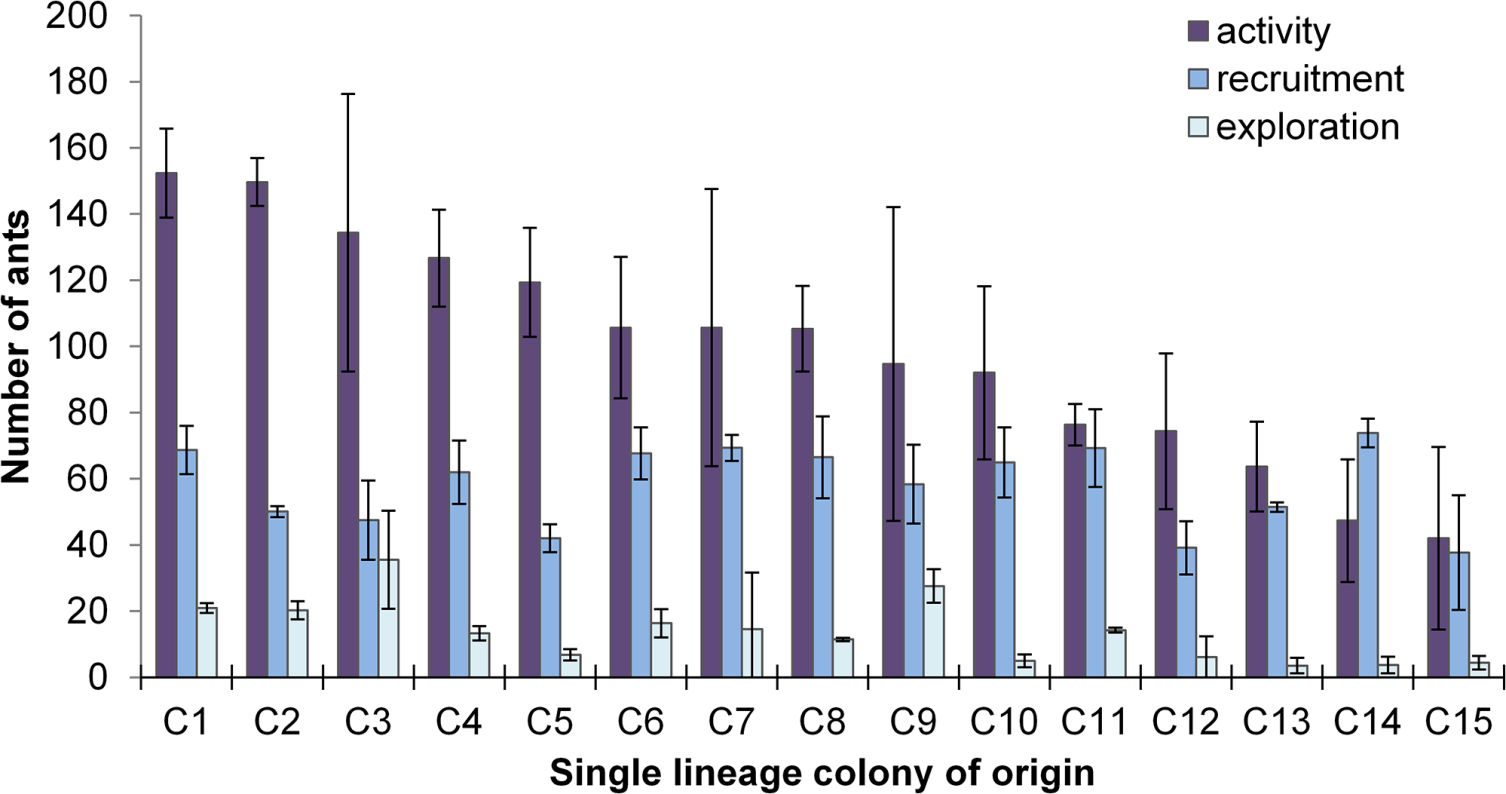
Foraging behavior of fire ants from different colony lineages. Graphs show average extra-nest activity (dark), recruitment to cricket between ten and forty minutes (medium), and exploratory activity (light) of each colony lineage (C1-C15, n = 3). Error bars show standard error.

When reared from single queens in standardized environments, worker lineage (single-lineage colony of origin) explained nearly half of the total observed behavioral variation among colonies for all measured traits (R^2^: exploratory activity = 49.65% extra-nest activity = 45.50%, recruitment = 45.48%). Groups of workers varied significantly in extra-nest activity and exploratory activity, and these differences were significantly affected by workers colony of origin (extra-nest: F_13,30_ = 2.15, p = 0.0414; exploratory: F_13,30_ = 3.25, p = 0.0038; recruitment F_13,30_ = 1.96; p = 0.0632; Fig 6). The R^2^ values provide an estimate of the broad-sense heritability of the traits.

## Discussion

The results of this study suggest that natural populations of fire ants exhibit substantial colony-level variation in foraging behavior both among and within populations, that this variation persists over time and across environments, and that this variation can have significant consequences for colony performance. Previous research on social insects has focused largely on behavioral variation within colonies [14,49,50] and studies specifically demonstrating variation in behavior among colonies are limited and are often snapshots in time [22,25,51,52]. We know of only a few that demonstrate persistence of variation across more than a few weeks [53,54]. In one of the best studies on ants to date, Bengston and Dornhaus [24] found colony-level variation in activity, aggression, and foraging effort in a cavity-dwelling ant *(Temnothorax rugatulus)*. They concluded that *T*. *rugatulus* colonies could be roughly classified as “risk-averse” and “risk-prone,” with risk-averse colonies combining high foraging effort with low aggression and shorter foraging distances. *T*. *rugatulus* colony activity levels did not correlate with other behaviors. We found that fire ant colonies also varied in their foraging effort (*e*.*g*. discovery time, trail formation, and recruitment), and higher foraging correlated with higher exploratory and extra-nest activity. Intriguingly, in our study increased foraging activity was associated with an increased amount of food collected by colonies, while increased extra-nest activity was associated with decreased colony growth, and these performance measures varied independently. Stable differences in the collective behavior of groups have been shown in a wide variety of organisms spanning various degrees of sociality [9,18,55,56]. The well-defined and obligate nature of eusocial groups makes ants and other eusocial arthropods an excellent model system for studying the evolution and effects of variation in collective behaviors. More studies are needed to discover the extent and effects of colony-level behavioral variation in social insects.

We found evidence higher activity levels may diminish colony growth rates under some conditions. The most active colonies in our study were as much as three times smaller at the end of our experiment than the least active colonies. Colony size is strongly correlated with the survival and performance of social insect colonies [57–59], suggesting that high activity colonies may pay a substantial fitness cost. Moreover, the persistence of differing behaviors we observed suggests colony behavior is not fully plastic and colonies “commit” to an activity level, at least over the timescales we observed. If higher activity is sometimes beneficial, colonies may face an “across-situation” trade-off between activity levels that maximize growth in different contexts [27]. For example, sticklebacks that exhibit consistently higher boldness and activity consume more food when competing with heterospecifics, but may have higher mortality when predator density is high [60]. In our experiment, colony growth may have been negatively affected by worker mortality associated with desiccation while foraging outside the nest, increased senescence associated with overworking, and/or worker allocation away from critical interior nest maintenance tasks (e.g., nursing and colony hygiene)[61]. Under field conditions, foraging workers would additionally face exposure to predators, competitors, and other hazards which could impose additional costs to activity outside the nest [32]. We predict, however, that more active colonies may ameliorate these costs in some field conditions where they may also be more likely to discover and dominate patchily distributed food resources and/or more effective at patrolling and controlling larger territories [62,63]. For example, in harvester ants, increased foraging activity resulted in higher reproductive success only when environmental conditions were poor [53]. In order to understand the evolution and maintenance of behavioral variation among colonies, future studies are needed that not only assess behavioral trade-offs across different contexts but also further explore the selective forces that produce them.

Our results demonstrate that colonies may express substantial behavioral variation independent of significant environmental variation during establishment and growth. We estimated a broad-sense heritability of between 0.45 and 0.5 for the foraging-related behaviors observed. Estimates of heritability of behavioral traits are rare among ants, but our results are comparable to the narrow-sense heritability estimates for worker and gyne mass in acorn ants, *Temnothorax* (*h* ^*2*^ = 0.37, 0.74), as well as for colony-level behavioral variation in pollen-hoarding behavior in honey bees (*h* ^*2*^ = 0.5) [64,65]. Although maternal and other environmental effects may be widespread in social insects, we think that the colony-level variation we observed likely has a genetic basis. In harvester ants, for example, daughter colonies resemble their mother colonies in the choice of days in which they reduce foraging activity [26]. If the level of broad-sense heritability we estimated even remotely reflects narrow-sense heritability (additive genetic variation among colonies), then it seems highly likely that fire ant foraging behavior could be under selection and evolving. Page and Fondrk [66] demonstrated that selection could alter pollen-hoarding behavior of honey bee colonies (*h* ^*2*^ = 0.5) within a single generation. Future studies should seek to identify genes that vary between colony lineages, and compare the behavior and fitness of these colonies under different environmental conditions and selective pressures.

The colony-level variation that we observed could have broad ecological consequences. Consistent intraspecific differences in behavior can mediate the magnitude and nature of species interactions [27]. For example, in funnel web spiders more aggressive individuals often prey on a wider range of organisms, while in trout more active individuals have higher encounter rates with both predators and prey [67,68]. We expect field colonies of fire ants with high or low patterns of foraging and activity to impact interacting species in consistently different ways. Ant foraging behavior is well known to be able to alter dominance hierarchies and diversity of competing ant communities, initiate both top-down and bottom-up trophic cascades, and change seed dispersal patterns of plants [12,69,70]. Our data indicate that these important ecological effects are likely to vary depending on the behavior of neighboring fire ant colonies. Incorporating measures of heredity and intraspecific trait variation has been found to significantly improve models of community assembly and alter predictions of extinction risk, population spread, and the outcomes of species interactions [3,71,72]. There is increasing interest in the ability of more heritable traits to shift the balance of evolution and ecological dynamics [3]. Higher heritability and increased intraspecific variation of ecologically important traits allows more rapid adaptation, increasing the chance of persistence in novel environments and decreasing the window of time in which a species may be displaced by better adapted competitors [73]. We expect that comparing the relative heritabilities of foraging behavior and other competitively important traits between ant species will increase accuracy of predictions of success and spread of invasive ants. Documenting colony-level variation in behaviors associated with the ecosystem functions provided by social insects will be critical to more accurately predict and potentially manage the ecological effects of these pervasive and critically important animals.

The regional variation we observed suggests that macro-environmental factors may affect colony-level behavioral variation. Throughout the experiment, colonies from the Texas site exhibited on average higher activity and recruitment, faster resource discovery, and greater weight of food collected compared to colonies from the Mississippi site. Texas colonies also tended to increase their extra-nest activity over time, while Mississippi colonies tended to reduce activity. Imported fire ants (species complex *S*. *invicta* and *S*. *richteri)* were introduced into Alabama more than 70 years ago and expanded through the coastal US, reaching Mississippi around 1940 and spreading into Texas around 1975 [74]. Intriguingly, our results align with the “spatial sorting” hypothesis, which predicts that faster or more active individuals will move further from the invasion origin, leading to assortative mating and the evolution of faster individuals at the spreading edge of an invasion [75]. Bengston and Dornhaus [24] found colony behavior of native *T*. *rugatulus* ants varied along a latitudinal gradient. It would be extremely interesting to test fire ant colony behavior patterns along an invasion gradient. Recent studies have hypothesized that behavioral syndromes, particularly those geared toward higher activity and aggression, may contribute to invasive success [76–79]. Comparisons across native and invasive range may help to clarify the role of evolution and changing environment in the success of invasions and the evolution of collective behavior.

Research has increasingly highlighted the importance of considering behavior across multiple contexts and organizational levels [9,80]. The results of this study lend new support to the idea that colony-level variation in social insect behavior is likely to be widespread in natural populations and can have significant consequences for colony performance which selection may act upon [9,81]. In addition, we take the first steps to address several important gaps in current knowledge and highlight important topics to be explored in the future. Very few studies have assessed the role of recent experience on colony-level personality. Furthermore, our results implicate a heritable component to the collective behavior of colonies and raise the possibility that colony-level traits may be diverging among populations due to selective pressures on activity and foraging behavior.

## Supporting Information

S1 Fig Diagram of novel climbing structure used to measured exploratory activity. Two halves of an index card were skewered vertically on a bamboo skewer.
(TIF)

S2 Fig Foraging habitat and regional effects on fire ant resource discovery and trail formation times. Graphs show average time to discovery for fire ants recruiting to (a) ground-level or (b) elevated foraging resources and average time to formation of a recruiting trail for (c) ground-level or (d) elevated foraging resources for standardized experimental colonies in standardized foraging habitats before and after being exposed to different foraging habitats for five weeks. Colonies are grouped by region of origin (Texas, light diamond vs. Mississippi, dark square). Error bars show standard error.
(TIF)

S1 Table Analysis of fire ant resource discovery and trail formation times before and after exposure to different foraging habitats. Table summarizes repeated measures analysis of variance for standardized experimental colonies in standardized foraging habitats before and after being exposed to different foraging habitats for five weeks. Within subjects effects use multivariate analysis of variance; lambda is converted to the appropriate F value. Asterisks denote significance at alpha = 0.05.
(PDF)
