# Supplementary figures and images for: Intraspecific Variation among Social Insect Colonies: Persistent Regional and Colony-Level Differences in Fire Ant Foraging Behavior
Source: PLoS One. 2015 Jul 21;10(7):e0133868. doi: 10.1371/journal.pone.0133868 (PMC4510567; doi:10.1371/journal.pone.0133868)

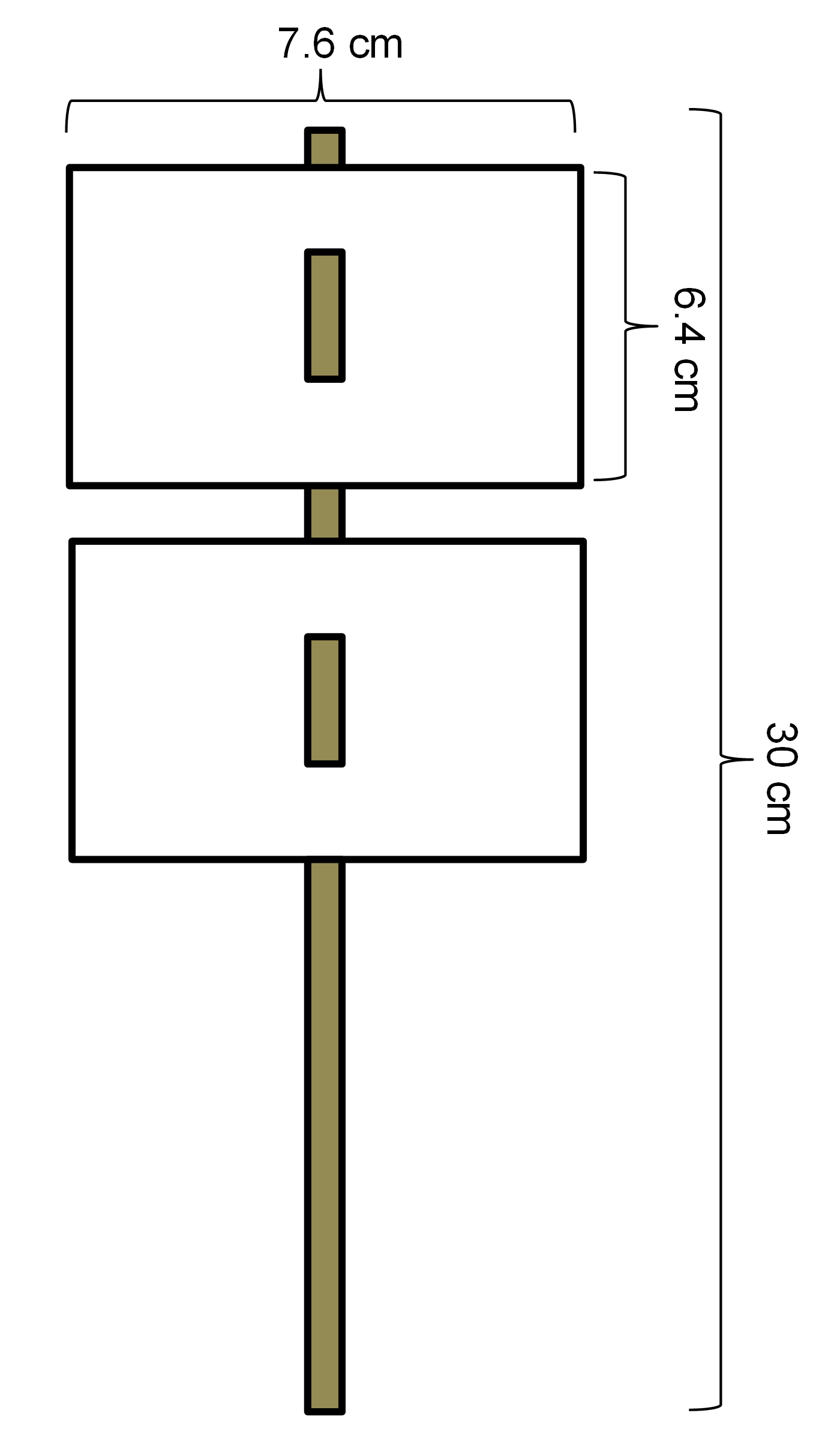

Supplement: S1 Fig — Two halves of an index card were skewered vertically on a bamboo skewer. (TIF) [file pone.0133868.s001.tif]

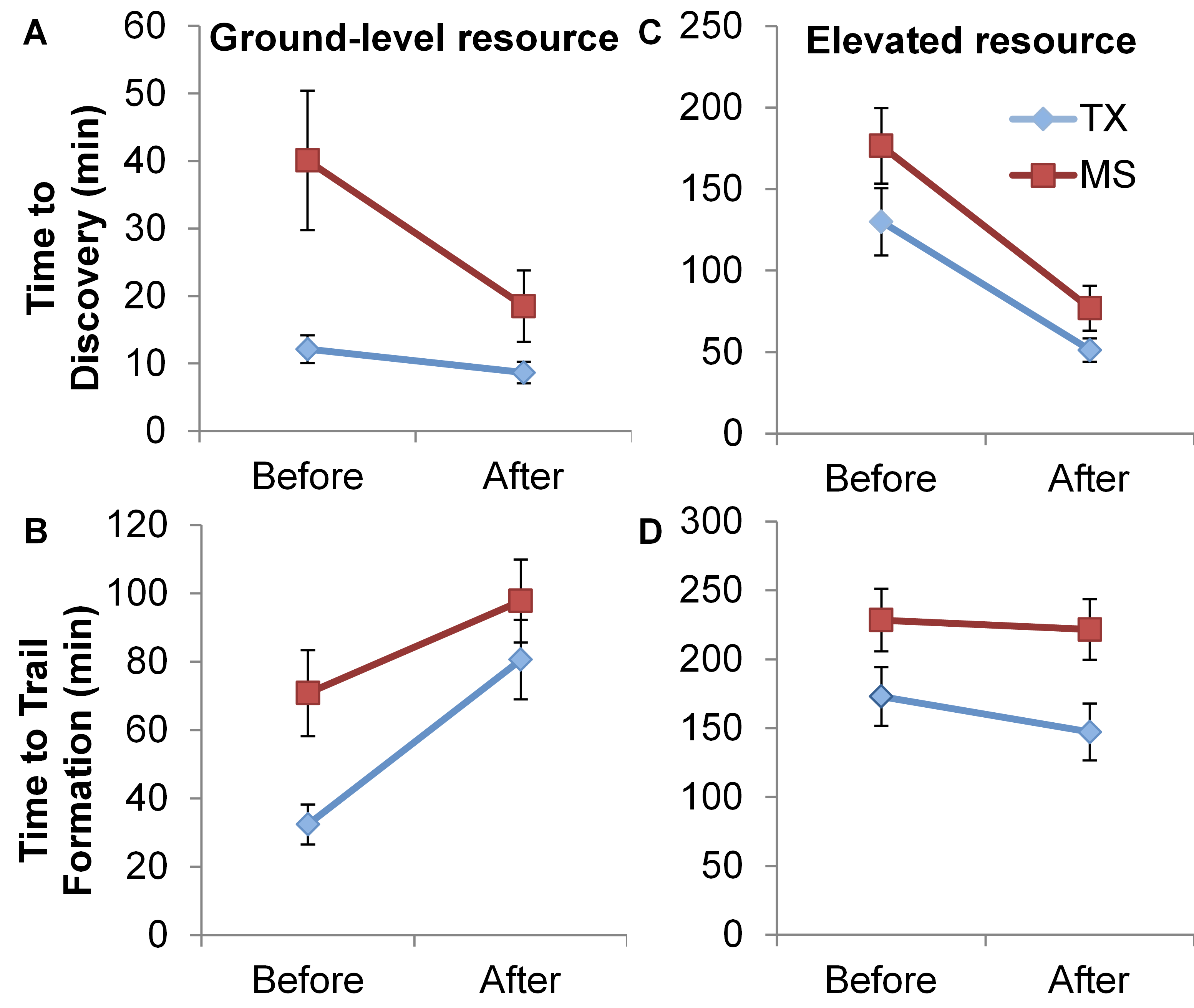

Supplement: S2 Fig — Graphs show average time to discovery for fire ants recruiting to (a) ground-level or (b) elevated foraging resources and average time to formation of a recruiting trail for (c) ground-level or (d) elevated foraging resources for standardized experimental colonies in standardized foraging habitats before and after being exposed to different foraging habitats for five weeks. Colonies are grouped by region of origin (Texas, light diamond vs. Mississippi, dark square). Error bars show standard error. (TIF) [file pone.0133868.s002.tif]
