# Supplementary material for: Intraspecific Variation among Social Insect Colonies: Persistent Regional and Colony-Level Differences in Fire Ant Foraging Behavior
Source: PLoS One. 2015 Jul 21;10(7):e0133868. doi: 10.1371/journal.pone.0133868 (PMC4510567; doi:10.1371/journal.pone.0133868)
Supplement: S1 Table — Table summarizes repeated measures analysis of variance for standardized experimental colonies in standardized foraging habitats before and after being exposed to different foraging habitats for five weeks. Within subjects effects use multivariate analysis of variance; lambda is converted to the appropriate F value. Asterisks denote significance at alpha = 0.05. (PDF) [file pone.0133868.s003.pdf]

S1 Table. Analysis of fire ant resource discovery and trail formation times before and after exposure to different foraging habitats.

Trait	Effect	p	F	df 1	df 2
**Discovery time (elevated)**	Foraging Habitat	0.6940	0.16	1	32
	Region	0.0058*	8.73	1	32
	Colony(Region)	0.0003*	3.60	31	32
	Time	<0.0001*	40.18	1	32
	Time*F.Habitat	0.6141	0.26	1	32
	Time*Region	0.4647	0.55	1	32
	Time*Colony(Region)	0.0734	1.69	31	32
**Discovery time (ground-level)**	Foraging Habitat	0.4479	0.59	1	32
	Region	0.0006*	14.45	1	32
	Colony(Region)	0.0582~	1.76	31	32
	Time	0.0661	3.62	1	32
	Time*F.Habitat	0.1047	2.79	1	32
	Time*Region	0.1782	1.89	1	32
	Time*Colony(Region)	0.9830	0.46	31	32
**Trail formation (elevated)**	Foraging Habitat	0.3007	1.11	1	32
	Region	0.0004*	15.49	1	32
	Colony(Region)	0.0015*	2.96	31	32
	Time	0.2861	1.18	1	32
	Time*F.Habitat	0.3383	0.95	1	32
	Time*Region	0.5353	0.39	1	32
	Time*Colony(Region)	0.0038*	2.65	31	32
**Trail formation (ground-level)**	Foraging Habitat	0.5015	0.46	1	32
	Region	0.0010*	13.20	1	32
	Colony(Region)	<0.0001*	4.29	31	32
	Time	<0.0001*	19.09	1	32
	Time*F.Habitat	0.5456	0.37	1	32
	Time*Region	0.2258	1.53	1	32
	Time*Colony(Region)	0.2766	1.24	31	32

Table summarizes repeated measures analysis of variance for standardized experimental colonies in standardized foraging habitats before and after being exposed to different foraging habitats for five weeks. Within subjects effects use multivariate analysis of variance; lambda is converted to the appropriate F value. Asterisks denote significance at $\alpha=0.05$.
